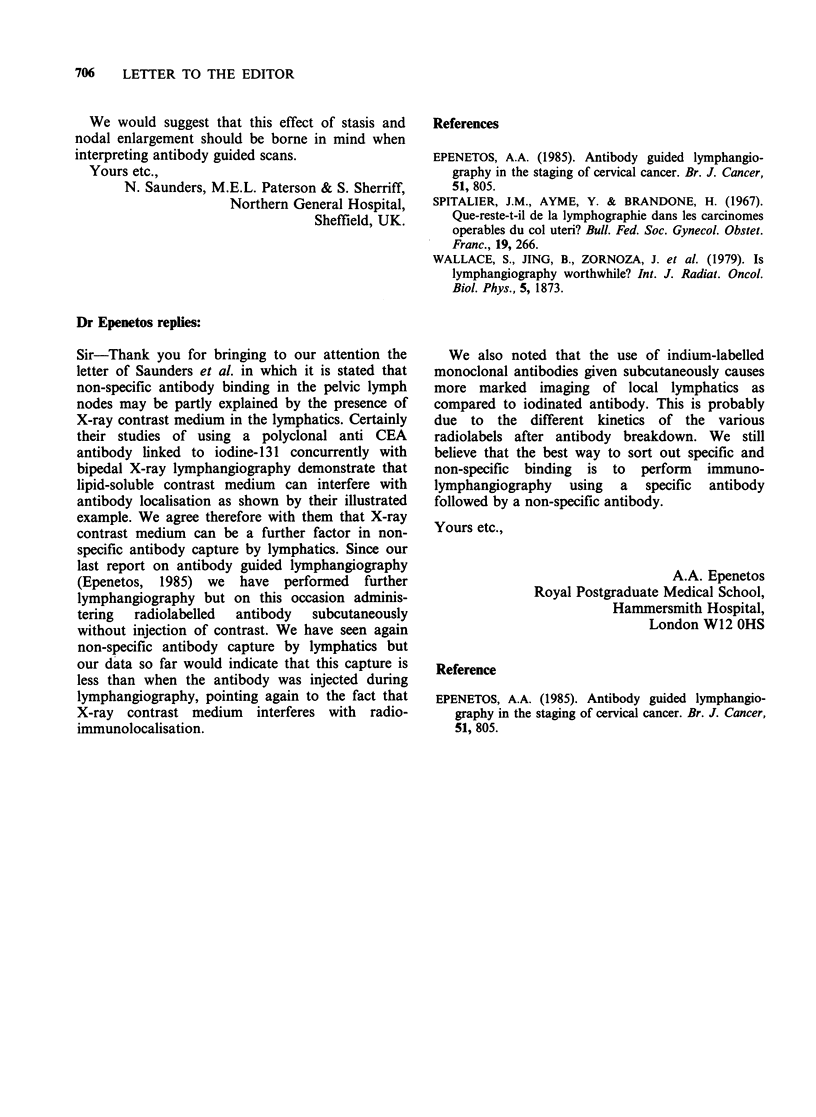# Dr Epenetos replies

**Published:** 1986-05

**Authors:** A.A. Epenetos


EPENETOS, A.A. (1985). Antibody guided lymphangiography in the staging of cervical cancer. *Br. J. Cancer*, **51**, 805.

SPITALIER, J.M., AYME, Y. & BRANDONE, H. (1967). Que-reste-t-il de la lymphographie dans les carcinomes operables du col uteri? *Bull. Fed. Soc. Gynecol. Obstet. Franc.*, **19**, 266.

WALLACE, S., JING, B., ZORNOZA, J. *et al.* (1979). Is lymphangiography worthwhile? *Int. J. Radiat. Oncol. Biol. Phys.*, **5**, 1873.

### Dr Epenetos replies:

Sir—Thank you for bringing to our attention the letter of Saunders *et al.* in which it is stated that non-specific antibody binding in the pelvic lymph nodes may be partly explained by the presence of X-ray contrast medium in the lymphatics. Certainly their studies of using a polyclonal anti CEA antibody linked to iodine-131 concurrently with bipedal X-ray lymphangiography demonstrate that lipid-soluble contrast medium can interfere with antibody localisation as shown by their illustrated example. We agree therefore with them that X-ray contrast medium can be a further factor in non-specific antibody capture by lymphatics. Since our last report on antibody guided lymphangiography (Epenetos, 1985) we have performed further lymphangiography but on this occasion administering radiolabelled antibody subcutaneously without injection of contrast. We have seen again non-specific antibody capture by lymphatics but our data so far would indicate that this capture is less than when the antibody was injected during lymphangiography, pointing again to the fact that X-ray contrast medium interferes with radioimmunolocalisation.

We also noted that the use of indium-labelled monoclonal antibodies given subcutaneously causes more marked imaging of local lymphatics as compared to iodinated antibody. This is probably due to the different kinetics of the various radiolabels after antibody breakdown. We still believe that the best way to sort out specific and non-specific binding is to perform immunolymphangiography using a specific antibody followed by a non-specific antibody.

Yours etc.,

A.A. Epenetos
Royal Postgraduate Medical School,
Hammersmith Hospital,
London W12 0HS

### Reference